# Cross-Layer Optimization for Heterogeneous MU-MIMO/OFDMA Networks

**DOI:** 10.3390/s21082744

**Published:** 2021-04-13

**Authors:** Kyu-haeng Lee, Daehee Kim

**Affiliations:** 1 Department of Mobile Systems Engineering, Dankook University, Yongin-si 16890, Korea; kyuhaeng.lee@dankook.ac.kr; 2 Department of Internet of Things, Soonchunhyang University, Asan-si 31538, Korea

**Keywords:** MU-MIMO, OFDMA, heterogeneous networks, cross-layer optimization

## Abstract

To enable the full benefits from MU-MIMO (Multiuser-Multiple Input Multiple Output) and OFDMA (Orthogonal Frequency Division Multiple Access) to be achieved, the optimal use of these two technologies for a given set of network resources has been investigated in a rich body of literature. However, most of these studies have focused either on maximizing the performance of only one of these schemes, or have considered both but only for single-hop networks, in which the effect of the interference between nodes is relatively limited, thus causing the network performance to be overestimated. In addition, the heterogeneity of the nodes has not been sufficiently considered, and in particular, the joint use of OFDMA and MU-MIMO has been assumed to be always available at all nodes. In this paper, we propose a cross-layer optimization framework that considers both OFDMA and MU-MIMO for heterogeneous wireless networks. Not only does our model assume that the nodes have different capabilities, in terms of bandwidth and the number of antennas, but it also supports practical use cases in which nodes can support either OFDMA or MU-MIMO, or both at the same time. Our optimization model carefully takes into account the interactions between the key elements of the physical layer to the network layer. In addition, we consider multi-hop networks, and capture the complicated interference relationships between nodes as well as multi-path routing via multi-user transmissions. We formulate the proposed model as a Mixed Integer Linear Programming (MILP) problem, and initially model the case in which each node can selectively use either OFDMA or MU-MIMO; we then extend this to scenarios in which they are jointly used. As a case study, we apply the proposed model to sum-rate maximization and max–min fair allocation, and verify through MATLAB numerical evaluations that it can take appropriate advantage of each technology for a given set of network resources. Based on the optimization results, we also observe that when the two technologies are jointly used, more multi-user transmissions are enabled thanks to flexible resource allocation, meaning that greater use of the link capacity is achieved.

## 1. Introduction

Remarkable advances in radio technology over the past few decades have ushered in a new era of wireless communications. IEEE 802.11ax [1], the newest Wi-Fi standard, is theoretically able to support a link rate of 11 Gbps with 160 MHz bandwidth and eight spatial streams, and 3GPP has aimed to provide a downlink peak data rate of 20 Gbps and latency of <1 ms for cellular systems since Release 15 [2]. These achievements can primarily be attributed to the two innovative technologies of OFDMA (Orthogonal Frequency Division Multiple Access) and MU-MIMO (Multiuser-Multiple Input Multiple Output). By dividing a transmission into multiple subchannels, OFDMA can fully use the available radio resources in both the time and frequency domains, and the network capacity can be greatly increased using MU-MIMO without requiring additional bandwidth. Moreover, these technologies enable multiple network entities to communicate simultaneously, which in turn offers great opportunities for enhancing the flexibility of wireless networking.

To benefit fully from the potential of these two technologies, researchers have long been interested in their optimal use under conditions of constrained network resources. With MU-MIMO, nodes can transmit or receive as many spatial streams as the number of antennas, and thus the DoF (Degree of Freedom) has been regarded as a key element in optimization [3,4,5,6,7], while in the field of OFDMA research, the appropriate scheduling of resource blocks (scheduling units consisting of a set of subcarriers) has been investigated for diverse network scenarios and objectives [8,9,10,11]. One valuable tool for optimal resource allocation is *cross-layer optimization* [3,4,5,6,7,8,9,10,11,12,13,14,15], as this can take advantage of the collaborative operation among the upper and lower layers of the traditional OSI (Open Systems Interconnection) network model, and thus help reduce the wastage of resources when exchanging information between them. In addition to MIMO/OFDMA networks, cross-layer optimization is actually being used for various network scenarios. For example, a reactive link layer acknowledgement mechanism has been proposed in which the transport layer is considered together to achieve high reliability of data delivery in IEEE 802.15.4/6LoWPAN networks [16]; an optimal multi-path routing method for wireless sensor networks is taken into account in conjunction with duty-cycle at MAC layer [17]; in network systems allowing for flexible and fine control over network elements, such as SDN (Software Defined Network), cross-layer optimization can be more effective [18,19].

Although various network scenarios and factors have been considered in previous studies, most of these have several limitations. First, a few studies have considered interactions between OFDMA [8,9,10,11] and MU-MIMO [3,4,5,6,7]; instead, most have focused on maximizing the performance of only one of these technologies. Several recent studies have addressed the joint use of these within optimization models, but have considered only single-hop networks [12,13,14,15,20,21]. In this network model, the effect of interference between nodes is relatively limited, which causes the network performance to be overestimated, and as a result, these approaches may not be applicable to various multi-hop network scenarios. The importance of research on multi-hop networks has been much emphasized thus far. There are a growing number of applications deployed on multi-hop networks, due to the desirable features, such as easy deployment and maintenance, and extended coverage via relay nodes. Above all, the multi-hop network enables a more comprehensive and fundamental understanding of communication technologies, so that the theoretical performance limits and characteristics of the network can be uncovered.

Secondly, even though the types and capabilities of network devices are becoming more diverse, this heterogeneity has not been sufficiently considered in relation to optimization; the same available bandwidth is assumed [13,14,15,20,21], or even the same number of antennas of nodes [14], and in particular, the joint use of OFDMA and MU-MIMO is generally assumed to be always available at all the nodes [13,14,15,20,21]. These assumptions may have the advantage of simplifying the model, but they are somewhat different from the conditions in actual networks; for example, although OFDMA has now been introduced to Wi-Fi systems through IEEE 802.11ax [1], the joint use of MU-MIMO and OFDMA is not allowed, and most legacy devices, including IoT (Internet of Things) products, are unable to operate with a wide band of 160 MHz and large number of antennas, due to the form factor and the processing power required. Regardless of how quickly high-capacity devices that fully use both technologies can penetrate the market, they will co-exist with low-capacity devices for a long time, and this heterogeneity should therefore be considered to allow for more practical and realistic resource allocation.

In this paper, we propose a cross-layer optimization framework that considers both OFDMA and MU-MIMO in heterogeneous wireless networks. Our approach assumes not only that nodes have different capabilities in terms of bandwidth and the number of antennas, but also supports practical use cases in which nodes can operate with either OFDMA or MU-MIMO, or both at the same time. The resultant resource allocation allows these technologies to be appropriately combined and exploited in a given network. In our optimization model, we carefully consider the interactions between the key elements of the physical layer to the network layer. In particular, we consider multi-hop networks, and capture the complicated interference relationships between nodes as well as multi-path routing via multi-user transmissions. We formulate the proposed model as a Mixed Integer Linear Programming (MILP) problem. Since there are a few devices that can support the combined use of OFDMA and MU-MIMO, we first model a case in which each node can selectively use either OFDMA or MU-MIMO. Next, we demonstrate that this model can be easily extended to scenarios where OFDMA and MU-MIMO are jointly used. As reported in many cross-layer optimization studies [9,10,12,13,14,15], one challenging issue when designing the model relates to the tightly coupled variables between layers, which are inherently nonlinear. We transform all the constraints of this nonlinear relationship into linear constraints, meaning that integer programming algorithms such as branch-and-bound can be easily applied to our model. In addition, we show that a little extra analysis of the given network can be of great help in reducing the size of the problem and thus making it more feasible. As a case study, we apply the proposed model to sum-rate maximization and max–min fair optimization, and verify through MATLAB numerical evaluations that it can take appropriate advantage of each technology for a given set of network resources. We also analyze how the joint use of MU-MIMO and OFDMA affects the results of resource allocation and the overall performance. From the optimization results, we can observe that when the two technologies are used in combination, more multi-user transmissions are enabled thanks to flexible resource allocation, hence giving better use of the link capacity.

The main contributions of this paper can be summarized as follows:

1. We propose a cross-layer optimization framework for heterogeneous MU-MIMO/OFDMA multi-hop networks. Our model takes into account the heterogeneity in the node capabilities in terms of the bandwidth, the number of antennas, and the modes of transmission. Moreover, the interactions between the key elements of the physical layer to the network layer for multi-hop networks are considered in the model.
2. We develop the proposed model using MILP, and formulate optimization models for two cases: one in which the node selectively uses either MU-MIMO or OFDMA, and one where it can use both at the same time. We also provide a method of reducing the problem size.
3. We verify the feasibility of the proposed model through MATLAB numerical evaluations. By applying it to two different optimization problems, we show that the proposed model can be effectively used for diverse network scenarios. The optimization results provide insight into how the joint use of MU-MIMO and OFDMA affects the overall performance.

The remainder of this paper is organized as follows. In Section 2, we summarize previous works related to this paper. Section 3 explains the network model, and the proposed optimization framework is described in Section 4. Section 5 presents the results of the MATLAB evaluations, and the paper is concluded in Section 6.

## 2. Related Work

Numerous studies have investigated ways of fully using the potential of OFDMA and MU-MIMO under a given set of network resources. In the OFDMA system, a resource block (known as a resource unit in Wi-Fi), composed of a set of subcarriers and with a fixed time length, is used as a basic unit for resource allocation. Many resource block allocation schemes have been proposed to satisfy various network requirements and objectives. Fathi et al. consider the joint optimization of the subcarrier and modulation rate in the downlink of wireless mesh networks [8]. Power and subcarrier allocation methods for uplink OFDMA networks, based on the user’s channel state, are also proposed in many studies in the literature [9,10], and the QoE (Quality of Experience) in the application layer is taken into account, in conjunction with the power and subcarrier allocation, in a work by Rugelj et al. [11]. In MU-MIMO, the maximum number of spatial streams that can theoretically be transmitted or received is the same as the number of antennas of a node, and thus the maximization of DoF use has attracted great attention [3,4,5,6,7]. The optimal link layer models developed by Hou and Blough et al. [3,4,7] carefully capture the conditions required for spatial multiplexing and interference cancellation to maximize the number of spatial streams in the network. Wang et al. investigate joint bandwidth allocation and DoF assignment [5], and Chu et al. explore integrated routing and MIMO link scheduling [6]. However, these studies are limited by the fact that they each focus on optimizing the performance of only one of these two technologies, as mentioned earlier.

Several recent works have begun to consider OFDMA and MU-MIMO together [12,13,14,15,20,21]. Femanias et al. examine subcarrier and power allocation as well as stream selection for downlink networks [12]. Along this line, Gangwar et al. propose an optimal subcarrier allocation algorithm that maximizes the total system capacity subject to the total transmit power minimization [22]. Hamda et al. optimize the transmit power in an uplink cooperative MU-MIMO/OFDMA network by taking into account optimal relay selection [13]. In addition, many resource allocation methods have been proposed for various purposes, for example user-fairness [20], QoS (Quality of Service) [21], and PSNR (Peak Signal-to-Noise Ratio) for video communications [14]. Similar to our work, the recent seminal work by Kordbacheh et al. models the interaction from the physical data rate to routing in the optimization for MU-MIMO/OFDMA networks, but a different approach is employed: greedy algorithm development from nonlinear optimization formulation [15]. Although these works consider both OFDMA and MU-MIMO, the main drawback is that they are only applicable to single-hop networks. In this scenario, as mentioned before, the effect of interference is limited, and thus the network performance may be overestimated, meaning that these models are impractical.

In addition, the heterogeneity of the nodes has not been sufficiently considered in these approaches; they are based on the assumption of equal available bandwidth [13,14,15,20,21] or the same number of antennas for the nodes [14], and in particular, the joint use of OFDMA and MU-MIMO is assumed to be always available at all the nodes [13,14,15,20,21]. Optimal resource allocation in MIMO/OFDMA heterogeneous networks in fact has attracted much attention, and various heterogeneity aspects have been investigated in recent studies. A work by Sakata et al. is motivated by the fact that concurrent transmission of data of different transmission times could make a big waste of transmission resources [23]. Different channel conditions and queue lengths of the nodes in downlink MIMO/OFDMA networks are taken into account in a work by Danobeitia [24]. Lee considers the heterogeneous maximum bandwidths of nodes in IEEE 802.11ax-based networks, and OFDMA resource allocation is formulated as a utility maximization problem in terms of the performance of both the uplink OFDMA and the downlink MU-MIMO [25]. Unfortunately, most of them are limited to single-hop networks, and the simultaneous use of OFMDA and MU-MIMO is also assumed.

## 3. Network Model and Assumptions

In this section, we present a network model and explain the assumptions made in this paper. Consider a heterogeneous wireless network with |N| nodes and |L| links, where *N* and *L* are sets of nodes and links, respectively. Each node i∈N has different communication capabilities in terms of the number of antennas (denoted as $S_{i}$) and the bandwidth (denoted as $B_{i}$). In this network, a total bandwidth of $B^{\max}$ is available, consisting of *K* subchannels, each of which has $B^{\min}$ bandwidth. We consider a total number of flows |F| in the network, where each flow f∈F is characterized by its source and destination nodes, denoted as $f^{\mathrm{src}}$ and $f^{\mathrm{dst}}$, respectively. When a data flow *f* passes through a link *l*, it is transmitted at a certain data rate, which is determined by the transmission mode and the allocated resources. More specifically, the number of spatial streams and the allocated bandwidth determine the data rate of flow *f* on link *l*. In this paper, we use a unit data rate in which one data stream with $B^{\min}$ bandwidth (i.e., one subchannel) corresponds to one unit data rate [3,4,7]. Based on this value, the actual data rate is computed as the product of the number of allocated spatial streams and the bandwidth. We assume that power allocation is properly conducted if the bandwidth is allocated. We use a scheduling scheme based on time slots: within a given time slot *t* (1≤t≤T), only a subset of links can be active. Uppercase notation is used to represent the model parameters, and lowercase for variables. In addition, for simplicity, it is assumed that the notation for the sets also indicates their size; for example, *N* is a set of nodes, and at the same time represents the number of nodes.

When a node is in communication, it can operate in one of two modes: OFDMA or MU-MIMO. Initially, we assume that the nodes cannot use both OFDMA and MU-MIMO at the same time, and in Section 4.4, we relax this restriction and show that the proposed model can be easily extended to the case of joint MU-MIMO and OFDMA optimization. If a node uses OFDMA, it uses only a single antenna, meaning that only a single spatial stream can be activated. Conversely, if it uses MU-MIMO, it can exploit multiple spatial streams, but these share the same network bandwidth, which generally requires a sufficient value for the channel bandwidth (e.g., at least 20 MHz [26,27]). We adopt different minimum bandwidth requirements for OFDMA and MU-MIMO, denoted as $B^{\min}$ and $B^{\left(\mathrm{MIMO}\text{-}\min\right)}$, respectively. Nodes can deal with multiple outgoing and incoming streams via OFDMA or MU-MIMO if the total bandwidth and the DoFs consumed by the outgoing/incoming streams do not exceed the given limits. In addition, we assume that streams with different transmission modes cannot be processed together.

**Figure 1:**
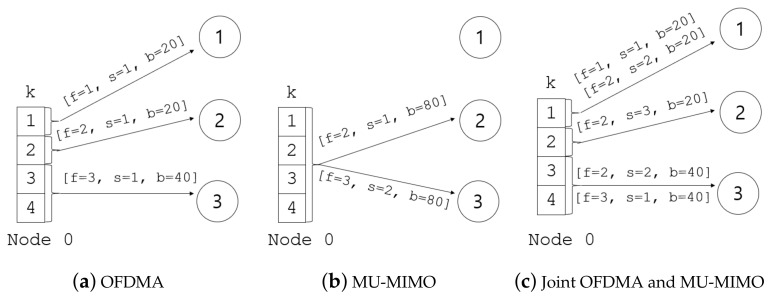
Comparison of OFDMA, MU-MIMO, and joint OFDMA and MU-MIMO operation, where *f*, *s* and *b* represent the index of the flow, the number of spatial streams and the bandwidth, respectively.

The illustrations in Figure 1 show three transmission modes: OFDMA, MU-MIMO, and joint OFDMA and MU-MIMO. In each figure, the four blocks on the left show the four subchannels, and *T* is set to one. For node 0 with three antennas that wants to transmit frames, Figure 1a shows an example of resource allocation when it chooses OFDMA. We can see that node 0 concurrently transmits three frames: for flow 1, via link 0 → 1; for flow 2, via link 0 → 2; and for flow 3, via link 0 → 3. All three OFDMA transmissions are conducted through a single spatial stream (and hence s=1), but with different bandwidths: 20 MHz for flows 1 and 2, and 40 MHz for flow 3. Assuming that $B^{\min}$ is 20, the sum-rate in this case is 4 (=1+1+2). We now turn to the case of MU-MIMO shown in Figure 1b. In this case, node 0 transmits two frames for flow 2 and flow 3 via links 0 → 2 and 0 → 3, respectively. Unlike in OFDMA, both frames are transmitted with the same bandwidth (i.e., b=80), but the numbers of allocated spatial streams are different. A single spatial stream is allocated to flow 2 on link 0 → 2, while two spatial streams are allocated to flow 3. In this case, the sum-rate becomes 12 (=4+8), which is larger than that for OFDMA. Figure 1c shows the case of joint use of MU-MIMO and OFDMA. In this case, each OFDMA transmission can have multiple spatial streams. For example, over subchannel 1, node 0 transmits two types of data to node 1: the data for flow 1 with one spatial stream, and the data for flow 2 with two spatial streams. In this case, the sum-rate is 12 (=3+3+6). Since the maximum bandwidth is 80 MHz (i.e., four subchannels) and the number of antennas for the transmitter is three, the maximum achievable capacity of the network in this example is 12, which is obtained both using MU-MIMO (Figure 1b) and OFDMA and MU-MIMO jointly (Figure 1c). Although this result suggests that the joint use of OFDMA and MU-MIMO does not give any advantage in terms of increasing the network capacity, and does not seem to be of much benefit, it enables a more flexible resource allocation than when only one of the two technologies is used, thereby helping increase the overall performance, as will be discussed later in Section 5.2.

## 4. Optimization Framework

In this section, we elaborate the proposed optimization framework in detail. We first explain the decision variables used in the model and constraints imposed on them in Section 4.1, and then add more constraints to model the OFDMA and MU-MIMO operations in Section 4.2. In Section 4.3, we discuss the complexity of the model and a method of reducing the model size, and we describe the extended model for the joint MU-MIMO and OFDMA case in Section 4.4.

### 4.1. Decision Variables

#### 4.1.1. Data Rate

We summarize the decision variables of the proposed model in Table 1. Let us begin with the model for the rate of flow. In our framework, the rate of flow *f*, denoted as $r_{f}$, is defined as the achievable end-to-end data rate of flow *f*, and similarly, $r_{lf}$ denotes the rate of flow *f* on link *l*. Then, from the property of flow conservation [3,6], we have the following constraints:

(1) $$\sum_{l\in L_{i}^{\mathrm{out}}}r_{lf}=r_{f},\;\;\;\;\forall f,i=f^{\mathrm{src}}$$

(2) $$\sum_{l\in L_{i}^{\mathrm{out}}}r_{lf}=\sum_{l\in L_{i}^{\mathrm{in}}}r_{lf},\;\;\;\;\forall f,i\neq f^{\mathrm{src}},f^{\mathrm{dst}}$$

(3) $$\sum_{l\in L_{i}^{\mathrm{in}}}r_{lf}=r_{f},\;\;\;\;\forall f,i=f^{\mathrm{dst}}$$

where $L_{i}^{\mathrm{out}}$ and $L_{i}^{\mathrm{in}}$ are the sets of outgoing links and incoming links for node *i*, respectively. Please note that constraint (3) is automatically met if constraints (1) and (2) are satisfied [3,6].

Since before reaching the destination, the data in flow *f* may pass through multiple links with different capacities, we need to consider the capacity of each individual link. As mentioned earlier, the capacity is related to the number of spatial streams and the bandwidth, and these may vary for each time slot depending on the scheduling result. To take these aspects into consideration, we define two variables, $z_{ltfs}$ and $c_{ltfs}$: for flow *f* on link *l* at time slot *t*, the binary variable $z_{ltfs}$ indicates whether or not the number of allocated spatial streams is *s*, and $c_{ltfs}$ is a continuous variable that denotes the corresponding capacity. Then, because the value of $r_{lf}$ cannot exceed the average capacity of link *l* for flow *f* over the whole time slot, we can impose the following constraint:

(4) $$r_{lf}\leq \frac{1}{T}\sum_{t,s}c_{ltfs},\;\;\;\;\forall l,f.$$

In addition, based on the definition of $c_{ltfs}$, we have:

(5) $$c_{ltfs}=b_{ltf}\cdot z_{ltfs},\;\;\;\;\forall l,t,f,s$$

where $b_{ltf}$ is the bandwidth allocated to flow *f* on link *l* at time slot *t*, which is computed as the total number of assigned subchannels. Let $z_{ltfk}$ be a binary variable that indicates whether or not subchannel *k* is allocated to flow *f* on link *l* at time slot *t*. Then, using $z_{ltfk}$, we can derive $b_{ltf}$ as follows:

(6) $$b_{ltf}=B^{\min}\sum_{k}z_{ltfk}\leq B_{l}^{\max}\cdot z_{ltf},\;\;\;\;\forall l,t,f$$

where the rightmost term means that the value of $b_{ltf}$ is limited to the maximum bandwidth of the link, denoted as $B_{l}^{\max}$. Here, $z_{ltf}$ is a binary variable indicating whether flow *f* is active on link *l* at time slot *t*. This value has the following relationship to the variables $z_{ltfs}$ and $z_{ltfk}$:

(7) $$z_{ltf}=\sum_{s}z_{ltfs}\leq 1,\;\;\;\;\forall l,t,f$$

(8) $$z_{ltf}=\max_{k}\left(z_{ltfk}\right),\;\;\;\;\forall l,t,f,k.$$

For the flow *f* on link *l* to be activated at time slot *t*, it should be allocated to at least one spatial stream (constraint (7)) and one subchannel (constraint (8)). Please note that constraint (8) is expressed in a nonlinear form, and we therefore convert it to the following linear constraint:

(9) $$z_{ltfk}\leq z_{ltf}\leq \sum_{k}z_{ltfk},\;\;\;\;\forall l,t,f,k.$$

Although the variables for the link capacity have now been defined, one remaining problem is that constraint (5) is expressed as the product of two variables, and is therefore inherently nonlinear. However, it can be also equivalently represented as a set of linear constraints, as follows:

(10) $$B^{\min}\cdot c_{ltfs}\leq s\cdot B_{l}^{\max}\cdot z_{ltfs},\;\;\;\;\forall l,t,f,s$$

(11) $$s\left(b_{ltf}-B_{l}^{\max}\cdot \left(1-z_{ltfs}\right)\right)\leq B^{\min}\cdot c_{ltfs}\leq s\cdot b_{ltf},\;\;\;\;\forall l,t,f,s.$$

Please note that $B^{\min}$ is multiplied with $c_{ltfs}$, since we assume a unit data rate, which is achieved when a single spatial stream (i.e., s=1) and a bandwidth of $B^{\min}$ are used.

#### 4.1.2. Transmission Mode

Thus far, we have defined the variables related to the data rate. In the following, we will discuss the variables related to MU-MIMO and OFDMA transmission modes. Since at this stage, we are assuming that nodes cannot use MU-MIMO and OFDMA at the same time, it is necessary to introduce some variables to indicate the transmission mode of the currently active link or node. To do this, we define several variables that begin with *m* and *o*. Two binary variables, $m_{ltf}$ and $o_{ltf}$, are used to indicate whether flow *f* on link *l* at time slot *t* is invoked in MU-MIMO mode or OFDMA mode, respectively. Since the nodes cannot activate MU-MIMO and OFDMA at the same time, we have the following constraint:

(12) $$m_{ltf}+o_{ltf}=z_{ltf},\;\;\;\;\forall l,t,f.$$

Recall that $z_{ltf}$ is already limited to one by constraint (7). Similarly, we introduce the following variables: $m_{it}^{x}$, $o_{it}^{x}$, $m_{it}^{y}$ and $o_{it}^{y}$, each of which indicates whether node *i* at time slot *t* is a MU-MIMO transmitter, an OFDMA transmitter, a MU-MIMO receiver, or an OFDMA receiver, respectively. Then, assuming the property of half-duplex transmission, we have:

(13) $$m_{it}^{x}+m_{it}^{y}+o_{it}^{x}+o_{it}^{y}\leq 1,\;\;\;\;\forall i,t.$$

We can readily see that these variables are defined using $m_{ltf}$ and $o_{ltf}$, as follows: $m_{it}^{x}=\max_{l\in L_{i}^{\mathrm{out}},f}\left(m_{ltf}\right)$, $m_{it}^{y}=\max_{l\in L_{i}^{\mathrm{in}},f}\left(m_{ltf}\right)$, $o_{it}^{x}=\max_{l\in L_{i}^{\mathrm{out}},f}\left(o_{ltf}\right)$, and $o_{it}^{y}=\max_{l\in L_{i}^{\mathrm{in}},f}\left(o_{ltf}\right)$. By employing the same technique used in constraint (9), we can convert them to the following linear constraints:

(14) $$m_{ltf}\leq m_{it}^{x}\leq \sum_{l,f}m_{ltf},\;\;\;\;\forall i,l\in L_{i}^{\mathrm{out}},t,f$$

(15) $$m_{ltf}\leq m_{it}^{y}\leq \sum_{l,f}m_{ltf},\;\;\;\;\forall i,l\in L_{i}^{\mathrm{in}},t,f$$

(16) $$o_{ltf}\leq o_{it}^{x}\leq \sum_{l,f}o_{ltf},\;\;\;\;\forall i,l\in L_{i}^{\mathrm{out}},t,f$$

(17) $$o_{ltf}\leq o_{it}^{y}\leq \sum_{l,f}o_{ltf},\;\;\;\;\forall i,l\in L_{i}^{\mathrm{in}},t,f.$$

In addition, to model the behavior of the node in each subchannel, we introduce the following set of binary variables: $m_{itk}^{x}$, $m_{itk}^{y}$, $o_{itk}^{x}$ and $o_{itk}^{y}$. Each of these indicates whether node *i* transmits or receives data over subchannel *k* at time slot *t* using MU-MIMO or OFDMA, respectively. Similarly, we can also express them in terms of the variables defined above; for example, $m_{itk}^{x}$ is equivalent to $\max_{l\in L_{i}^{\mathrm{out}},f}\left(z_{ltfk}\right)\;\&\&\;m_{it}^{x}$. The relevant constraints are given below:

(18) $$m_{itk}^{x}\leq m_{it}^{x},\;\;\;\;\forall i,t,k$$

(19) $$m_{itk}^{y}\leq m_{it}^{y},\;\;\;\;\forall i,t,k$$

(20) $$o_{itk}^{x}\leq o_{it}^{x},\;\;\;\;\forall i,t,k$$

(21) $$o_{itk}^{y}\leq o_{it}^{y},\;\;\;\;\forall i,t,k$$

(22) $$z_{ltfk}+m_{it}^{x}-1\leq m_{itk}^{x}\leq \sum_{l\in L_{i}^{\mathrm{out}},f}z_{ltfk},\;\;\;\;\forall i,l\in L_{i}^{\mathrm{out}},t,f,k$$

(23) $$z_{ltfk}+m_{it}^{y}-1\leq m_{itk}^{y}\leq \sum_{l\in L_{i}^{\mathrm{in}},f}z_{ltfk},\;\;\;\;\forall i,l\in L_{i}^{\mathrm{in}},t,f,k$$

(24) $$z_{ltfk}+o_{it}^{x}-1\leq o_{itk}^{x}\leq \sum_{l\in L_{i}^{\mathrm{out}},f}z_{ltfk},\;\;\;\;\forall i,l\in L_{i}^{\mathrm{out}},t,f,k$$

(25) $$z_{ltfk}+o_{it}^{y}-1\leq o_{itk}^{y}\leq \sum_{l\in L_{i}^{\mathrm{in}},f}z_{ltfk},\;\;\;\;\forall i,l\in L_{i}^{\mathrm{in}},t,f,k.$$

From the assumption that streams of different transmission modes cannot be processed together, we have the following constraints:

(26) $$m_{itk}^{y}+o_{jtk}^{x}\leq 1,\;\;\;\;\forall i,j\in N_{i}^{\mathrm{int}},t,k$$

(27) $$o_{itk}^{y}+m_{jtk}^{x}\leq 1,\;\;\;\;\forall i,j\in N_{i}^{\mathrm{int}},t,k$$

where $N_{i}^{\mathrm{int}}$ is a set of nodes within the interference range of node *i*.

The remaining variables, $s_{itk}^{x}$ and $s_{itk}^{y}$, indicate the number of spatial streams allocated to node *i* in subchannel *k* at time slot *t*, and will be discussed in the following subsection.

### 4.2. Constraints on MU-MIMO and OFDMA Operation

In this subsection, we explain the constraints on the operations related to MU-MIMO and OFDMA transmission.

#### 4.2.1. Number of Spatial Streams

Let us first define $s_{ltf}$ as the number of spatial streams allocated to flow *f* on link *l* at time slot *t*. We can easily see that this is derived using $z_{ltfs}$, as follows: $s_{ltf}=\sum_{s}s\cdot z_{ltfs}$. In the case of MU-MIMO, it is clear that the total number of spatial streams on a link cannot exceed the maximum number of spatial streams available on that link, while for OFDMA case, $s_{ltf}$ is simply bounded at one. We can then combine these constraints into one, as follows:

(28) $$m_{ltf}+o_{ltf}\leq s_{ltf}\leq S_{l}^{\max}\cdot m_{ltf}+o_{ltf},\;\;\;\;\forall l,t,f$$

where $S_{l}^{\max}$ is the maximum number of spatial streams for link *l*, and determined as $\min(S_{i},S_{j})$, where *i* and *j* are the source and destination for link *l*, respectively.

We now discuss the constraints on $s_{itk}^{x}$ and $s_{itk}^{y}$. First, in the case of OFDMA, since the number of spatial streams is limited to one, the existing variables $o_{itk}^{x}$ and $o_{itk}^{y}$ can be used as they are to denote this. We will therefore regard the variables $s_{itk}^{x}$ and $s_{itk}^{y}$ here as the number of spatial streams when MU-MIMO is used. Then, from the constraint on the maximum number of spatial streams, we have:

(29) $$\sum_{l\in L_{i}^{\mathrm{out}},f}z_{ltfk}\leq s_{itk}^{x}\leq o_{itk}^{x}+S_{i}\cdot m_{itk}^{x},\;\;\;\;\forall i,t,k$$

(30) $$\sum_{l\in L_{i}^{\mathrm{in}},f}z_{ltfk}\leq s_{itk}^{y}\leq o_{itk}^{y}+S_{i}\cdot m_{itk}^{y},\;\;\;\;\forall i,t,k.$$

To model the requirement that all spatial streams of the same MU-MIMO transmission share the same bandwidth, we use the idea that the number of spatial streams allocated to each subchannel should be the same: that is, $s_{itk}^{x}=\sum_{l\in L_{i}^{\mathrm{out}},f}s_{ltf}$ and $s_{itk}^{y}=\sum_{l\in L_{i}^{\mathrm{in}},f}s_{ltf}$. This gives the following constraints:

(31) $$-U\left(1-m_{itk}^{x}\right)\leq s_{itk}^{x}-\sum_{l\in L_{i}^{\mathrm{out}},f}s_{ltf}\leq U\left(1-m_{itk}^{x}\right),\;\;\;\;\forall i,t,k$$

(32) $$-U\left(1-m_{itk}^{y}\right)\leq s_{itk}^{y}-\sum_{l\in L_{i}^{\mathrm{in}},f}s_{ltf}\leq U\left(1-m_{itk}^{y}\right),\;\;\;\;\forall i,t,k$$

where *U* is a large constant that ensures the constraints are valid. Please note that these constraints are relaxed in the case of joint MU-MIMO and OFDMA operation, which will be discussed later.

We also need to consider the effect on interfering streams. In the case of OFDMA, interfering streams can only be handled by allocating different channels, while in the case of MU-MIMO, even interfering streams coming through the same channel can be handled, if there exist remaining DoFs:

(33) $$\sum_{j\in N_{i}^{\mathrm{int}}}s_{jtk}^{x}\leq S_{i}\cdot m_{itk}^{y}+U\left(1-m_{itk}^{y}\right),\;\;\;\;\forall i,t,k$$

(34) $$\sum_{j\in N_{i}^{\mathrm{int}}}o_{jtk}^{x}\leq o_{itk}^{y}+U\left(1-o_{itk}^{y}\right),\;\;\;\;\forall i,t,k.$$

It is worth noting that *U* in the above constraints plays the same role as *U* in constraints (31) and (32), but their values do not have to be the same. Considering the other parameters given, such as *N* and *L*, it is possible to determine the *U* values for each constraint, which in turn helps to reduce the search space. Since it is straightforward to find appropriate *U* values for each constraint, this part will be omitted for simplicity in this paper.

#### 4.2.2. Bandwidth

The total bandwidth allocated to node *i* at time slot *t* cannot exceed the maximum available bandwidth (i.e., $B_{i}$). Due to the different minimum bandwidth requirements for MU-MIMO and OFDMA, we also have the following constraint:

(35) $$\begin{array}{cc} B^{\left(\mathrm{MIMO}\text{-}\min\right)}\cdot \left(m_{it}^{x}+m_{it}^{y}\right)+B^{\min}\cdot \left(o_{it}^{x}+o_{it}^{y}\right) & \leq B_{i}^{\min}\sum_{k}\left(m_{itk}^{x}+m_{itk}^{y}+o_{itk}^{x}+o_{itk}^{y}\right)\\ \; & \leq B_{i}\cdot \left(m_{it}^{x}+m_{it}^{y}+o_{it}^{x}+o_{it}^{y}\right),\;\;\;\forall i,t. \end{array}$$

In addition, since the range of subchannels allocated to the nodes cannot exceed the available bandwidths of the nodes, we have:

(36) $$B^{\min}\cdot \left(k_{1}-k_{2}+1\right)-B_{i}\leq U\cdot \left(2-m_{itk_{1}}^{x}-m_{itk_{2}}^{x}\right),\;\;\;\;\forall i,t,\left(1\leq k_{1},k_{2}\leq K\right)$$

(37) $$B^{\min}\cdot \left(k_{1}-k_{2}+1\right)-B_{i}\leq U\cdot \left(2-m_{itk_{1}}^{y}-m_{itk_{2}}^{y}\right),\;\;\;\;\forall i,t,\left(1\leq k_{1},k_{2}\leq K\right)$$

(38) $$B^{\min}\cdot \left(k_{1}-k_{2}+1\right)-B_{i}\leq U\cdot \left(2-o_{itk_{1}}^{x}-o_{itk_{2}}^{x}\right),\;\;\;\;\forall i,t,\left(1\leq k_{1},k_{2}\leq K\right)$$

(39) $$B^{\min}\cdot \left(k_{1}-k_{2}+1\right)-B_{i}\leq U\cdot \left(2-o_{itk_{1}}^{y}-o_{itk_{2}}^{y}\right),\;\;\;\;\forall i,t,\left(1\leq k_{1},k_{2}\leq K\right)$$

where $k_{1}$ and $k_{2}$ are subchannel indices and $k_{2}\leq k_{1}$. Please note that we can combine the above constraints into one since $m_{itk}^{x}+m_{itk}^{y}+o_{itk}^{x}+o_{itk}^{y}\leq 1$ is guaranteed.

Lastly, based on the assumption that MU-MIMO spatial streams should share the same network bandwidth, we have:

(40) $$\begin{array}{cc} -\left(2-m_{l_{1}tf}-m_{l_{2}tf}\right)\leq z_{l_{1}tfk}-z_{l_{2}tfk} & \leq (2-m_{l_{1}tf}-m_{l_{2}tf}),\\ & \;\;\;\;\;\;\;\;\;\;\;\;\forall i,l_{1}\in L_{i}^{\mathrm{out}}\cup L_{i}^{\mathrm{in}},l_{2}\in L_{i}^{\mathrm{out}}\cup L_{i}^{\mathrm{in}},t,f,k \end{array}$$

where $l_{1}$ and $l_{2}$ represent different links belonging to the same link set (i.e., $L_{i}^{\mathrm{out}}$ and $L_{i}^{\mathrm{in}}$).

### 4.3. Reducing the Model Size

Although the proposed model is a MILP problem, which is known to be NP-hard [28,29], several methods for obtaining optimal solutions and their implementations have already been developed. A description of the possible methods of solving a MILP problem is beyond the scope of this paper, but we briefly explain the general concept. MILP problems are typically solved using a branch-and-bound approach [29], in which the key idea is to repeatedly solve several LP (Linear Programming)-relaxations of the original problem until the optimal solution is found. We can generate LP relaxation problems by removing all the integer restrictions on the original problem. For example, let us call the original problem P0. A new problem obtained by removing all integer constraints from P0 now becomes an LP problem, and we can easily solve it with conventional convex optimization techniques. If the solution satisfies all the constraints of P0, then it is an optimal solution, so we can stop; otherwise, we create another LP problem and try to solve it again. More specifically, in this step, we pick a variable that is restricted to an integer, but whose value in the LP relaxation is fractional, and then generate two subproblems, P1 and P2, by imposing two different constraints on that variable. After computing optimal solutions for both P1 and P2, we can take the better of these two solutions. This process is repeated until we obtain the optimal solution.

Several techniques have been proposed to accelerate the performance of the branch-and-bound approach, such as presolving, cutting planes and heuristics [29]. Most of these techniques aim to limit the size of the search space that needs to be explored by intelligently removing duplicated constraints and unnecessary branches. It is also extremely valuable to do a little extra work on the problem to reduce the number of constraints. In our case, we can do this by pre-computing the candidate routing paths for the given flows. We introduce a parameter $Q_{lf}$, which indicates whether link *l* may belong to the possible routing paths of flow *f*. By using $Q_{lf}$, we not only narrow the lower and upper bounds on the decision variables, but also exclude unnecessary nodes and links from the optimization, so that we can further reduce the overall problem size. For example, the upper bound on the four-dimensional binary variables $z_{ltfs}$ and $z_{ltfk}$ can be more tightly determined using $Q_{lf}$, rather than simply set to one. We can also apply this approach to the constraints in the same way: for example, in constraint (4), if we multiply $Q_{lf}$ by the right side of the constraint, then some of the $r_{lf}$ values quickly become zero:

(41) $$r_{lf}\leq \frac{1}{T}\sum_{t,s}Q_{lf}\cdot c_{ltfs},\;\;\;\;\forall l,f.$$

**Figure 2:**
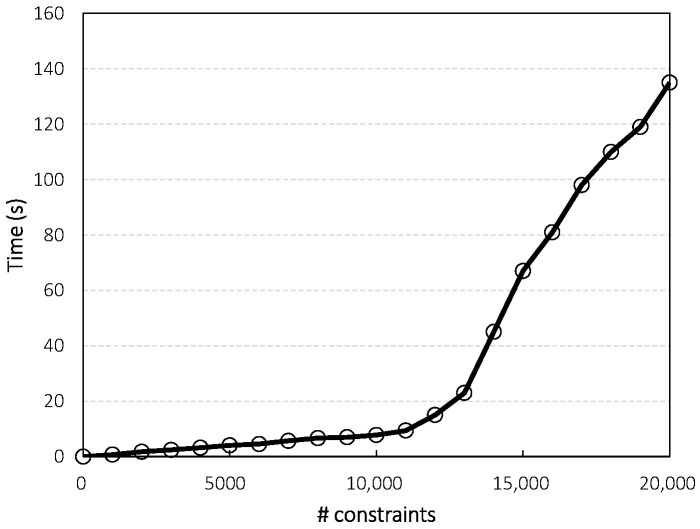
Average computation times for varying problem sizes.

$Q_{lf}$ can be computed with a simple DFS (Depth-First Search)-based algorithm, as shown in Algorithm 1. Please note that in line 16, we limit the maximum number of hops in the routing paths, so that unsuitable routing path candidates can be excluded from the optimization. Figure 2 shows the average computation time needed to obtain the optimal solution when $Q_{lf}$ is applied, for a network size represented in terms of several constraints. We performed experiments by varying the model parameters, such as *N*, *T*, and *F*, and taking 100 measurements of the computation time for each parameter set. Please note that even with the same parameters, the network is generated randomly each time, meaning that the number of constraints also changes. We divided a range of the number of constraints into several equal intervals, as shown in Figure 2, and calculated the average value for each interval. The measurements were conducted in the experimental environment described in Section 5.1. From the results, we can see that it takes less than 5 s until the number of constraints is lower than 5000, which is the value that can be obtained when *N* is around 15. However, the time required increases rapidly with the problem size, as expected, reaching about 140 s when the number of constraints is about 20,000, which is obtained when N=80, T=3, F=5, $S^{\max}=4$, and $B^{\max}=160$, where $S^{\max}$ is the maximum number of node antennas.

Although the use of the $Q_{lf}$ value can reduce the problem size to some extent, the high complexity of some variables and constraints still limits the scalability of the model. To alleviate this, we may need to further restrict the range of variables, or to relax some restrictions. For example, constraint (33) is originally imposed because the MU-MIMO receiver can theoretically handle interfering streams coming into the same channel, but for this transmission to actually be successful, a process for obtaining the channel information for the interfering nodes is required, which creates an excessive overhead [30]. Hence, rather than just focusing on maximizing the DoF, the allocation of potential interference streams to different channels would be more practical and plausible. Thus far, we have assumed that a single entity (e.g., central controller) solves the whole optimization problem, which might be infeasible for large-scale networks, but this assumption could be relaxed in several ways. One approach is to use local optimization with multiple dedicated nodes. We can further control the complexity by limiting the number of nodes or flows that each of them must serve. In this case, it is difficult to obtain global optimization, but this approach may be more appropriate to deal with dynamic network changes. Another method is to replace it with heuristic algorithms that separate coupled optimization elements so that a near-optimal solution can be obtained with low complexity, as adopted in many studies. We leave further study of this aspect to future work.
**Algorithm 1**$Q_{lf}$ Computation.1:$Q_{lf}\leftarrow \emptyset$2:G←(N,L)3:**for** f∈F **do**4:    $V\leftarrow f^{\mathrm{dst}},f^{\mathrm{src}}$               ▹*V* keeps track of the visited nodes.5:    P←∅                     ▹*P* is the path traced so far.6:    DFS$\left(f,G,V,P,Q_{lf}\right)$7:**end for**8:return $Q_{lf}$  9:**function** DFS($f,G,V,P,Q_{lf}$)10:    i←V(end)  ▹V(end) is the last visited node, while V(1) is the destination of *f*.11:    **for** $l\in L_{i}^{\mathrm{out}}$ **do**12:        **if** $l^{\mathrm{dst}}==V\left(1\right)$ **then**13:           $Q_{lf}\leftarrow 1$14:           ret←115:        **else**16:           **if** $l^{\mathrm{dst}}\notin V$ && |P|<h **then**       ▹*h* is the maximum number of hops.17:               $V\leftarrow V+l^{\mathrm{dst}}$18:               P←P+l19:               $\left(ret^{tmp},Q_{lf}\right)\leftarrow \mathrm{DFS}\left(f,G,V,P,Q_{lf}\right)$20:               $Q_{lf}\leftarrow ret^{tmp}$21:               $ret\leftarrow ret^{tmp}\left|\right|ret$22:               $V\leftarrow V-l^{\mathrm{dst}}$23:               P←P−l24:           **end if**25:        **end if**26:    **end for**27:    return ret, $Q_{lf}$28:**end function**

### 4.4. Joint Use of MU-MIMO and OFDMA

Thus far, we have assumed that the nodes cannot operate via MU-MIMO and OFDMA at the same time. In this subsection, we relax this restriction, and extend the model to joint MU-MIMO and OFDMA optimization. To achieve this, several changes must be made to the original problem. First, since OFDMA and MU-MIMO are now being used at the same time, we do not need to use the variables separating each transmission mode, such as $m_{ltf}$ and $o_{ltf}$. We leave only the OFDMA-related variables (i.e., variables starting with *o*), and redefine their meanings to include MU-MIMO operations. Variables related to MU-MIMO (i.e., variables starting with *m*) are removed from the constraints accordingly. In addition, several constraints such as (26), (27) and (40) are excluded, since they are no longer needed under these conditions. The following constraints are slightly modified compared to the original ones:

(42) $$\begin{array}{cc} o_{ltf}\leq s_{ltf}\leq S_{l}^{\max}\cdot o_{ltf}, & \;\;\;\;\;\;\;\;\;\;\;\;\forall l,t,f \end{array}$$

(43) $$\begin{array}{cc} \;s_{itk}^{x}\leq S_{i}\cdot o_{itk}^{x}, & \;\;\;\;\;\;\;\;\;\;\;\;\forall i,t,k \end{array}$$

(44) $$\begin{array}{cc} \;s_{itk}^{y}\leq S_{i}\cdot o_{itk}^{y}, & \;\;\;\;\;\;\;\;\;\;\;\;\forall i,t,k \end{array}$$

(45) $$\begin{array}{cc} \;\sum_{j\in N_{i}^{\mathrm{int}}}s_{jtk}^{x}\leq S_{i}\cdot o_{itk}^{y}+U\cdot \left(1-o_{itk}^{y}\right), & \;\;\;\;\;\;\;\;\;\;\;\;\forall i,t,k \end{array}$$

(46) $$\begin{array}{cc} \;-S_{l}^{\max}\cdot \left(1-z_{ltfs}\right)\leq s_{ltfk}-s\cdot z_{ltfk}\leq S_{l}^{\max}\cdot \left(1-z_{ltfs}\right), & \;\;\;\;\;\;\;\;\;\;\;\;\forall l,t,f,s,k. \end{array}$$

Based on the fact that OFDMA transmissions can now have multiple spatial streams, constraints (28)–(30) and (33) are replaced with constraints (42)–(45). Constraint (46) is newly added to represent $s_{itk}$, rather than constraints (31) and (32). Since different numbers of spatial streams in different subchannels are now available, we introduce variable $s_{ltfk}$ to denote the number of spatial streams for flow *f* on link *l* over subchannel *k* at time slot *t*.

## 5. Application Examples

### 5.1. Settings

In this section, we demonstrate the feasibility of the proposed model based on two optimization problems. We first consider a sum-rate maximization problem for which the objective is given below, and then discuss max–min fair allocation, in the following section:

(47) $$\begin{array}{c} \max\;\sum_{f\in F}r_{f}. \end{array}$$

The model parameters are given in Table 2. We randomly locate 10 nodes within a space of size 400 × 400 (unit: $m^{2}$), and the numbers of antennas (i.e., $S_{i}$) and the available bandwidth (i.e., $B_{i}$) of the nodes are set randomly, as shown in Table 3. The number of flows is set to two, and the source and destination nodes of each flow are selected randomly from the nodes. The data range and interference range are set to 200 and 300 m, respectively. We use a MATLAB optimization solver on an Intel i9 machine with 16 GB RAM, and before running the solver, we compute $Q_{lf}$ and apply it to the model to reduce the complexity. For a given network, we perform optimizations for four cases depending on the transmission mode as follows:

1. Case 1: the nodes selectively use either MU-MIMO or OFDMA.
2. Case 2: the nodes can use both at the same time.
3. Case 3: the nodes use only MU-MIMO.
4. Case 4: the nodes use only OFDMA.

Please note that Cases 3 and 4 are added as a baseline, since they could make more sense to current wireless communication systems than Case 1 and Case 2. For example, in the latest Wi-Fi standard, 802.11ax [1], multi-user transmissions using OFDMA and MU-MIMO, corresponding to Cases 1 and 2, are theoretically available, but it might be difficult for a node to change its transmission mode without any restrictions since their actual access mechanisms are totally different [31]. In addition, for some networks with low-capability devices, it is obviously more practical to consider Case 3 and Case 4.

**Table 2:** Model Parameters.

Parameter	Value
*N*	10
*F*	2
*T*	3
*K*	8
$S^{\max}$	4
$B^{\max}$	160
$B^{\min}$	20
$B^{\left(\mathrm{MIMO}\text{-}\min\right)}$	20
*U*	500

**Table 3:** Node Configurations for Sum-rate Maximization.

*i*	$S_{i}$	$B_{i}$
3	2	40
4	2	20
5	1	40
7	1	40
8	1	20
9	3	40
10	3	20

### 5.2. Numerical Results

**Figure 3:**
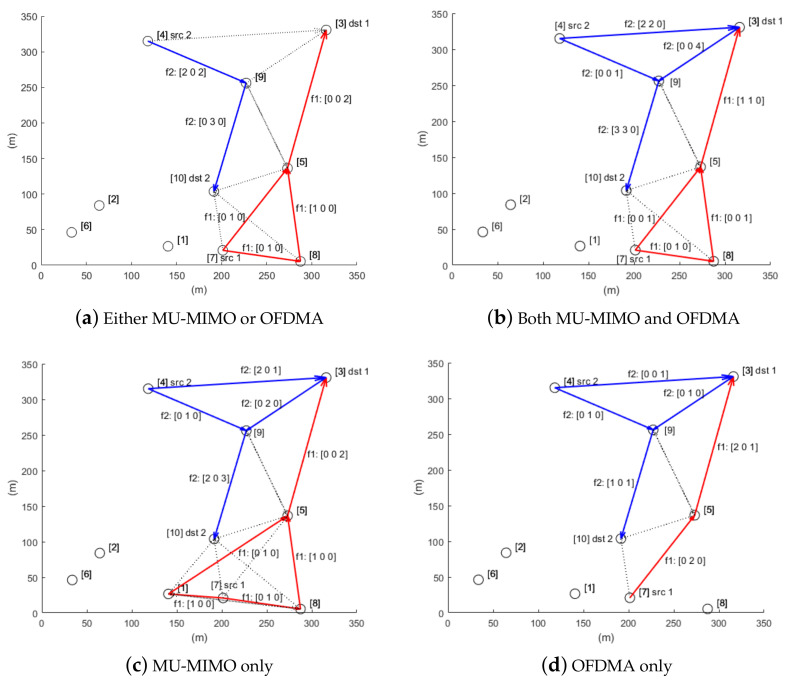
Resource allocation results for sum-rate maximization. The joint use of MU-MIMO and OFDMA (**b**) creates more multi-user transmissions, resulting in a higher sum-rate.

We illustrate the optimal solution in each case in Figure 3. In the figures, the red and blue arrows indicate the allocated links for flows 1 and 2, respectively, and the dotted arrows show the interference. The three values displayed next to the link refer to the resulting data rates of the link in each time slot. In particular, detailed resource allocation results for Cases 1 and 2 are given in Table 4 and Table 5.

First, we can see that multi-path routing is activated in all cases. In flow 1, node 7 is the source of flow 1 and is supposed to transmit data to nodes 5 and 8 with a unit data rate. In particular, in Case 1, these transmissions are conducted over OFDMA with a total bandwidth of 40 MHz, as shown in Table 4. From the fact that the maximum available bandwidth of node 7 is 40 MHz, as shown in Table 3, we can see that it fully uses OFDMA for these transmissions. Although all the resources allocated to flow 1 are OFDMA in Case 1, the transmissions for flow 2 are allocated to MU-MIMO resources. Links 4→9 and 9→10 are assigned two and three spatial streams, respectively. The outcome that all links for the same flow use an identical transmission method is not intentional, but is the result of chance. Recall that the proposed model allows a node to switch between transmission modes in different time slots. We assume that the reason OFDMA is preferred for flow 1 in Case 1 is because most of the nodes on the path of flow 1, i.e., nodes 5, 7 and 8, have only a single antenna.

The results for Case 2 are almost the same as those for Case 1, except that more links are allocated for flow 2. This is because a more flexible resource allocation is available in Case 2, which results in an increased number of multi-user transmissions. In Case 1, multi-user transmission occurs only at node 7, whereas in Case 2, multi-user transmissions are used at nodes 3, 5 and 9. More specifically, they differ in terms of the number of spatial streams allocated to each subchannel in OFDMA transmission. For example, nodes 4 and 5 simultaneously transmit data to node 3 over two time slots, using two spatial streams and one spatial stream over different subchannels. As another example, node 9 handles three incoming streams over subchannel 8: one spatial stream from node 4, and two spatial streams from node 3. Since the number of antennas for node 9 is three, this transmission is valid. This flexible resource allocation due to the joint use of MU-MIMO and OFDMA helps improve performance; according to the results, the sum-rate for Case 1 is 5/3 (=2/3+1), and for Case 2 is 7/3 (=2/3+5/3), i.e., a larger value than in Case 1.

Since multiple links are activated at the same time, they may suffer from interference; however, as can be seen from the results, most potential interference issues are resolved by allocating different subchannels. From the results for Case 2, we can identify several cases in which multiple links use the same subchannel, but these do not experience any interference issues: link 7→8 and link 4→3 are assigned to subchannel 6 at time slot 2, but they are out of range of interference with each other, and link 3→9 and link 4→9, which use the same subchannel 8 at time slot 3, are valid MU-MIMO transmissions, not interference.

Figure 3c,d depict the resource allocation results for the two cases where the nodes are restricted to using either one of two technologies. As expected, these results are quite different from those for previous two cases. For example, in Case 3, node 7 does not transmit to node 5 directly, different to the other three cases. In Case 4, all transmissions of flow 1 and flow 2 use a single spatial stream since MU-MIMO is not available. Please note that the reason the two links for flow 1 (links 7→5 and 5→3) have a data rate of 2 is because they are assigned to multiple subchannels. Such a difference, as a result, affects the overall performance: the sum-rate for Case 3 (MIMO only) is 5/3 (=2/3 + 1) and that for Case 4 (OFDMA only) is 4/3 (=2/3 + 2/3) which is the smallest value. More specifically, the data rate for flow 2 is reduced since the nodes of flow 2 (i.e., nodes 3, 4 and 9) cannot take advantage of MU-MIMO anymore in Case 4. This result is consistent with the fact that MU-MIMO is of much benefit in terms of increasing the network capacity as mentioned in Section 3.

### 5.3. Max–Min Fair Allocation

As mentioned earlier, the proposed model can be applied to various scenarios and objectives. In this section, we consider another optimization problem, a max–min fair allocation, whose objective function is defined as follows:

(48) $$\begin{array}{c} \max\;\min_{f\in F}r_{f}. \end{array}$$

The above objective is inherently nonlinear, and thus it is difficult to directly employ it in the proposed model. To address this issue, we introduce an additional auxiliary variable, denoted as *w*, add a constraint, and change the objective function as follows:

(49) $$\begin{array}{c} \max\;w \end{array}$$

(50) $$\begin{array}{cc} \;w\leq r_{f}, & \;\;\;\;\;\;\;\;\;\;\;\;\forall f. \end{array}$$

**Figure 4:**
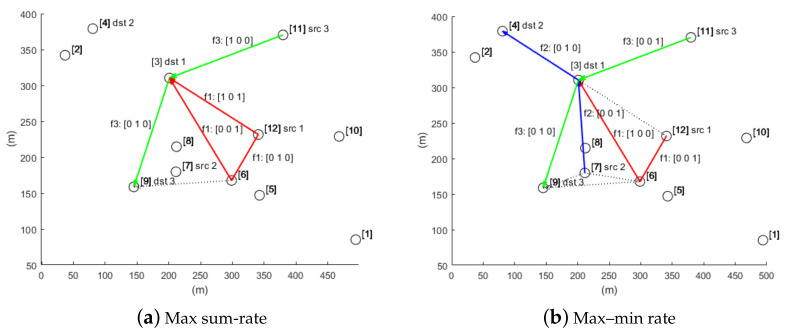
Resource allocation results for (**a**) the sum-rate maximization and (**b**) the max–min fair allocation. The max–min rate optimization achieves better fair resource allocation, at the expense of the sum-rate.

In this evaluation, the nodes are set to selectively use MIMO or OFDMA, and we use the same configuration of the previous evaluation except that N=12 and F=3, as shown in Table 6. Figure 4 compares two resource allocation results, and Table 7 and Table 8 show the corresponding detailed resource allocation results. First, we can clearly observe that flow 2 (i.e., blue arrows) is served only in the max–min fair allocation (Figure 4b). This allows for better fair allocation, but it also has performance degradation in terms of the sum-rate: with the sum-rate maximization the sum-rate is 4/3 (= 1 + 0 + 1/3), while with the max–min fair allocation, it is reduced to 1 (= 1/3 + 1/3 + 1/3). On the other hand, for the first case, relatively more resources are allocated to flow 1, resulting in a higher sum-rate, but no resources could be given to flow 2. Overall, the max–min fair allocation exploits OFDMA more than MIMO, compared to the sum-rate maximization case. OFDMA is used for all transmissions associated with flow 2. Node 3 fully uses its available bandwidth for OFDMA on links 3→4 and 3→9 at t=2, and for receiving data from two different sources, node 7 and node 11, at t=3. We also can see that most potential interference issues are resolved by allocating different subchannels, similar to the previous evaluation.

## 6. Conclusions

In this paper, we propose a cross-layer optimization framework that considers both OFDMA and MU-MIMO for heterogeneous wireless networks. Our model not only assumes that nodes have different capabilities in terms of their bandwidth and numbers of antennas, but also supports practical use cases in which nodes can operate with either OFDMA or MU-MIMO, or with both at the same time. By taking into account the interactions between the key elements of the physical layer to the network layer for multi-hop networks, we formulate the proposed model as a MILP problem, and through MATLAB numerical evaluations, we verify that it can take full advantage of each technology for a given set of network resources. In addition, from the optimization results, we can observe that when the two technologies are used jointly, more multi-user transmissions are enabled thanks to the flexibility of resource allocation, and thus higher use of the link capacity is achieved.

## Figures and Tables

**Table 1:** Decision Variables.

Variable	Description
$r_{f}$	rate of flow *f*
$r_{lf}$	rate of flow *f* on link *l*
$c_{ltfs}$	capacity of flow *f* on link *l* at time slot *t*, when *s* spatial streams are allocated
$z_{ltfs}$	indicates whether *s* spatial streams are allocated to flow *f* on link *l* at time slot *t*
$z_{ltfk}$	indicates whether subchannel *k* is allocated to flow *f* on link *l* at time slot *t*
$m_{ltf}$, $o_{ltf}$	indicate whether flow *f* on link *l* at time slot *t* is activated in MU-MIMO/OFDMA mode
$m_{it}^{x}$, $m_{it}^{y}$	indicate whether node *i* is in MU-MIMO transmission/reception mode at time slot *t*
$o_{it}^{x}$, $o_{it}^{y}$	indicate whether node *i* is in OFDMA transmission/reception mode at time slot *t*
$m_{itk}^{x}$, $m_{itk}^{y}$	indicate whether node *i* is in MU-MIMO transmission/reception mode in subchannel *k* at time slot *t*
$o_{itk}^{x}$, $o_{itk}^{y}$	indicate whether node *i* is in OFDMA transmission/reception mode in subchannel *k* at time slot *t*
$s_{itk}^{x}$, $s_{itk}^{y}$	indicate the number of outgoing/incoming spatial streams of node *i* in channel *k* at time slot *t*

**Table 4:** Resource Allocation Result for Case 1.

Link	t=1				t=2				t=3				Rate
	*f*	$s_{\mathrm{ltf}}$	$b_{\mathrm{ltf}}\left(k\right)$	Mode	*f*	$s_{\mathrm{ltf}}$	$b_{\mathrm{ltf}}\left(k\right)$	Mode	*f*	$s_{\mathrm{ltf}}$	$b_{\mathrm{ltf}}\left(k\right)$	Mode	
5 → 3	-	-	-	-	-	-	-	-	1	1	40 (2, 8)	OFDMA	[0 0 2]
7 → 5	-	-	-	-	1	1	20 (3)	OFDMA	-	-	-	-	[0 1 0]
7 → 8	-	-	-	-	1	1	20 (2)	OFDMA	-	-	-	-	[0 1 0]
8 → 5	1	1	20 (1)	OFDMA	-	-	-	-	-	-	-	-	[1 0 0]
4 → 9	2	2	20 (5)	MIMO	-	-	-	-	2	2	20 (3)	MIMO	[2 0 2]
9 → 10	-	-	-	-	2	3	20 (4)	MIMO	-	-	-	-	[0 3 0]

**Table 5:** Resource Allocation Result for Case 2.

Link	t=1			t=2			t=3			Rate
	*f*	$s_{\mathrm{ltf}}$	$b_{\mathrm{ltf}}\left(k\right)$	*f*	$s_{\mathrm{ltf}}$	$b_{\mathrm{ltf}}\left(k\right)$	*f*	$s_{\mathrm{ltf}}$	$b_{\mathrm{ltf}}\left(k\right)$	
5 → 3	1	1	20 (8)	1	1	20 (5)	-	-	-	[1 1 0]
7 → 5	-	-	-	-	-	-	1	1	20 (3)	[0 0 1]
7 → 8	-	-	-	1	1	20 (6)	-	-	-	[0 1 0]
8 → 5	-	-	-	-	-	-	1	1	20 (2)	[0 0 1]
3 → 9	-	-	-	-	-	-	2	2	40 (7, 8)	[0 0 4]
4 → 3	2	2	20 (7)	2	2	20 (6)	-	-	-	[2 2 0]
4 → 9	-	-	-	-	-	-	2	1	20 (8)	[0 0 1]
9 → 10	2	3	20 (6)	2	3	20 (7)	-	-	-	[3 3 0]

**Table 6:** Node Configurations for Max–Min Fair Allocation.

*i*	$S_{i}$	$B_{i}$
3	1	40
4	4	40
6	2	40
7	3	80
9	3	160
11	3	20
12	1	20

**Table 7:** Resource Allocation Result for the Sum-rate Maximization.

Link	t=1				t=2				t=3				Rate
	*f*	$s_{\mathrm{ltf}}$	$b_{\mathrm{ltf}}\left(k\right)$	Mode	*f*	$s_{\mathrm{ltf}}$	$b_{\mathrm{ltf}}\left(k\right)$	Mode	*f*	$s_{\mathrm{ltf}}$	$b_{\mathrm{ltf}}\left(k\right)$	Mode	
12 → 3	1	1	20(4)	OFDMA	-	-	-	-	1	1	20(6)	OFDMA	[1 0 1]
12 → 6	-	-	-	-	1	1	20(3)	MIMO	-	-	-	-	[0 1 0]
6 → 3	-	-	-	-	-	-	-	-	1	1	20(7)	OFDMA	[0 0 1]
11 → 3	3	1	20(5)	OFDMA	-	-	-	-	-	-	-	-	[1 0 0]
3 → 9	-	-	-	-	3	1	20(7)	MIMO	-	-	-	-	[0 1 0]

**Table 8:** Resource Allocation Result for the Max–Min Fair Allocation.

Link	t=1				t=2				t=3				Rate
	*f*	$s_{\mathrm{ltf}}$	$b_{\mathrm{ltf}}\left(k\right)$	Mode	*f*	$s_{\mathrm{ltf}}$	$b_{\mathrm{ltf}}\left(k\right)$	Mode	*f*	$s_{\mathrm{ltf}}$	$b_{\mathrm{ltf}}\left(k\right)$	Mode	
12 → 6	-	-	-	-	-	-	-	-	1	1	20(5)	OFDMA	[0 0 1]
6 → 3	1	1	20(6)	MIMO	-	-	-	-	-	-	-	-	[1 0 0]
7 → 3	-	-	-	-	-	-	-	-	2	1	20(4)	OFDMA	[0 0 1]
3 → 4	-	-	-	-	2	1	20(6)	OFDMA	-	-	-	-	[0 1 0]
11 → 3	-	-	-	-	-	-	-	-	3	1	20(3)	OFDMA	[0 0 1]
3 → 9	-	-	-	-	3	1	20(7)	OFDMA	-	-	-	-	[0 1 0]
